# C5aR deficiency attenuates the breast cancer development via the p38/p21 axis

**DOI:** 10.18632/aging.103468

**Published:** 2020-07-15

**Authors:** Jian Chen, Zi-han Sun, Li-ying Chen, Feng Xu, Yun-pei Zhao, Gui-qing Li, Ming Tang, You Li, Quan-you Zheng, Shu-feng Wang, Xin-hua Yang, Yu-zhang Wu, Gui-lian Xu

**Affiliations:** 1 Department of Immunology, Army Medical University (Third Military Medical University), Chongqing 400038, China; 2 Breast Disease Center, Guiqian International General Hospital, Guiyang 550000, China; 3 Institute of Cancer, Xinqiao Hospital, Army Medical University (Third Military Medical University), Chongqing 400037, China; 4 Urinary Nephropathy Center, The Second Affiliated Hospital of Chongqing Medical University, Chongqing 400065, China; 5 Department of ICU, Daping Hospital, Army Medical University (Third Military Medical University), Chongqing 400038, China; 6 Department of Urology, 958 Hospital, Army Medical University (Third Military Medical University), Chongqing 400020, China

**Keywords:** breast cancer, C5a/C5aR, p38, p21

## Abstract

Emerging evidence has shown activation of the complement component C5 to C5a in cancer tissues and C5aR expression in breast cancer cells relates to the tumor development and poor prognosis, suggesting the involvement of complement C5a/C5aR pathway in the breast cancer pathogenesis. In this study, we found that as compared to the non-tumoral tissues, both C5aR and MAPK/p38 showed an elevated expression, but p21/p-p21 showed lower expression, in the tumoral tissues of breast cancer patients. Mice deficient in C5aR or mice treated with the C5aR antagonist exhibited attenuation of breast cancer growth and reduction in the p38/p-p38 expression, but increase in p21/p-p21 expression, in the tumor tissues. Pre-treatment of the breast cancer cells with recombinant C5a resulted in reduced p21 expression, and MAPK/p38 inhibitors prevented C5a-induced reduction in p21 expression, suggesting the involvement of the MAPK/p38 signaling pathway in the C5a/C5aR-mediated suppression of p21/p-p21 expression. These results provide evidence that breast cancer development may rely on C5a/C5aR interaction, for which MAPK/p38 pathway participate in down-regulating the p21 expression. Inhibition of C5a/C5aR pathway is expected to be helpful for the treatment of patients with breast cancer.

## INTRODUCTION

Breast cancer (BC) is reported to be the most common cancer among women worldwide [1, 2]. Although there are numerous therapeutic strategies, including chemotherapy and radiotherapy as well as hormone and immune therapy, the mortality resulting from BC is the second leading cause of cancer deaths among women [3, 4]. The underlying mechanism is complex. Recent evidence shows that the tumor microenvironment, including inflammatory cells and molecules, plays a critical role in the tumor progression [5, 6].

Complement system, a vital component of the innate immune system, plays an important role in the immune-related inflammatory diseases [7]. Complement activation occurs in response to infection and a set of innate molecules. Once activated, the complement cascade generates a number of effector molecules, resulting in the cleavage of C3 and C5 to their activated forms, C3a and C5a, respectively and the terminal product C5b-9 [8]. C5a, acts as an inflammatory mediator when binding to its receptor, C5aR [9, 10]. It has been previously reported that the complement system contributes to cancer progression. Higher C5a levels were found in the lung cancer cell lines than in the nonmalignant lung epithelial cells [11]. The expression of C5aR was upregulated in various human cancer tissues, and BC C5aR expression was related to tumor development and poor prognosis of the patients [12, 13]. C5aR facilitated BC metastasis by altering T-Cell responses in the metastatic niche [14].

Controlling the cell cycle is a crucial process to regulate cell proliferation and apoptosis, especially in tumor cells [15]. P21 is a tumor suppressor that mediates cell cycle arrest at G1 or G2 phase. It was previously reported that the MAPK signal pathway regulates p21 transcription through transcription factors [16, 17]. C5aR activates the PI3K/AKT and MAPK signaling pathways and potentiates cell growth [18]. C5a/C5aR signaling contributes to the pathogenesis of some malignant tumors, such as gastric, thyroid, and lung cancer [11, 19–21]. However, the underlying mechanisms of C5a/C5aR signaling-mediated BC development remains unclear. In this study, our data indicate that the C5a/C5aR pathway promotes BC development via activation of the MAPK/p38 pathway.

## RESULTS

### Upregulation of C5aR expression in the tumoral tissues of BC patients

**Figure 1:**
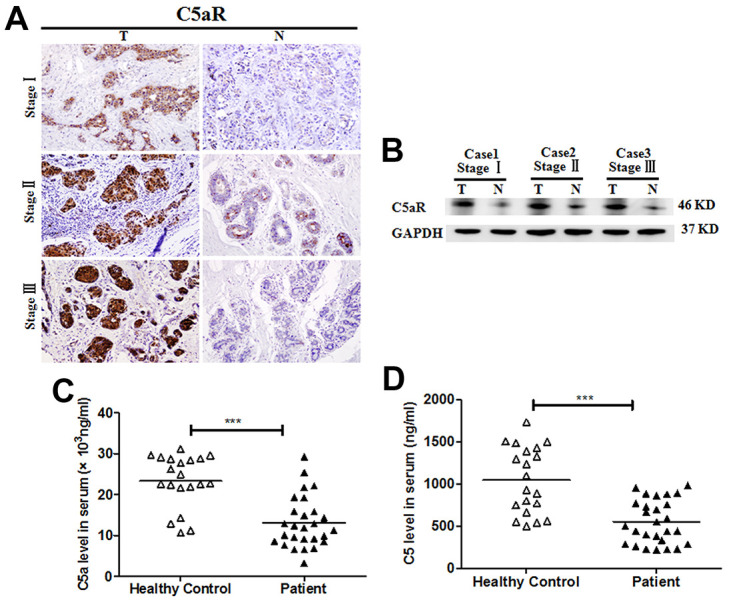
**Enhanced C5aR expression in tumoral tissues of patients with BC.** C5aR expression in BC tumoral tissues (T) and BC-adjacent non-tumoral tissues (N) was detected by (**A**) immunochemistry and (**B**) western blotting. (**C**) C5a and (**D**) C5 levels in serum were measured by enzyme-linked immunosorbent assay (ELISA) (healthy volunteers, n=20; BC patients, n=27). Magnification: 200×. The data are representative of three independent experiments. Data are presented as means ± SD. ***P<0.001.

To investigate the potential role of the C5a/C5aR pathway in BC, we analyzed C5aR expression in the tumoral tissues from BC patients at different clinical stages using IHC and western blotting. C5aR expression was significantly higher in the tumoral tissues than in the BC-adjacent non-tumoral tissues (Figure 1A, 1B). However, the C5a and C5 serum levels of patients with BC (n=27) exhibited a remarkable reduction, compared to healthy volunteers (n=20) (C5a: *P*<0.001, Figure 1C; C5: *P*<0.001, Figure 1D). The drop in serum C5a and C5 levels detected in the advanced BC patients was possibly due to the excessive activation and consumption of the complement in the early stages.

### Mice deficient in C5aR or treated with C5aR antagonists (C5aRa) exhibit significant attenuation of BC cell growth

**Figure 2:**
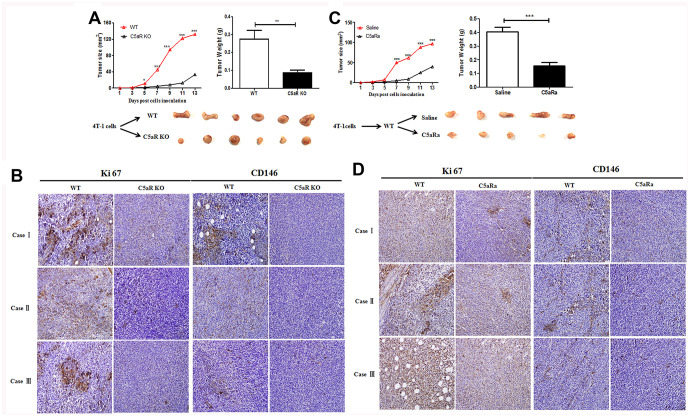
**Reduced tumor growth in mice with C5aR deficiency or C5aR signaling blockade.** Mice were received the murine BC cell line 4T-1 cells transplantation for tumor development. Tumor size was measured every 2 days. Mice were sacrificed in the indicated time and tumors tissues were collected. (C5aRa treatment: WT mice were injected with C5aRa 30 min prior to 4T-1 cells transplantation. Saline was used as the control of C5aRa). (**A**) Size and weight of tumors from WT and C5aR KO mice. (**B**) Ki67 and CD146 expression in WT and C5aRKO mice. (**C**) Size and weight of tumors from WT mice pretreated with Saline or C5aRa. (**D**) Ki67 and CD146 expression in WT mice with C5aRa and saline. (n = 5-6/group). Magnification: 200×. Three independent experiments were performed. *P < 0.05, **P < 0.01, ***P < 0.001.

To verify the effects of the C5aR signaling on BC development *in vivo*, mice were transplanted with the BC cell line 4T-1 cells (C5aR positive) (Supplementary Figure 1). C5aR deficiency caused a significant reduction in the tumor growth (Figure 2A). Moreover, the expression of Ki67 and CD146, which are closely related to the cell proliferation and angiogenesis, was remarkably downregulated in C5aR deficient mice (Figure 2B). Similar results were found when the mice were treated with the C5aRa (Figure 2C, 2D), a C5aR antagonist hexapeptide AcF (OP (D) ChaWR) [22]. In addition, the senescence of 4T-1 cells *in vitro* was significantly increased after C5aRa treatment (Supplementary Figure 2) while the invasion and metastasis of a non-adherent BC cell line MDA-MB-453 cells were accelerated after exposure to recombinant C5a (Supplementary Figure 3). All these results indicate the critical role of C5a/C5aR pathway in the pathogenesis of BC development.

### Downregulated p21 expression in tumor tissues of BC patients

**Figure 3:**
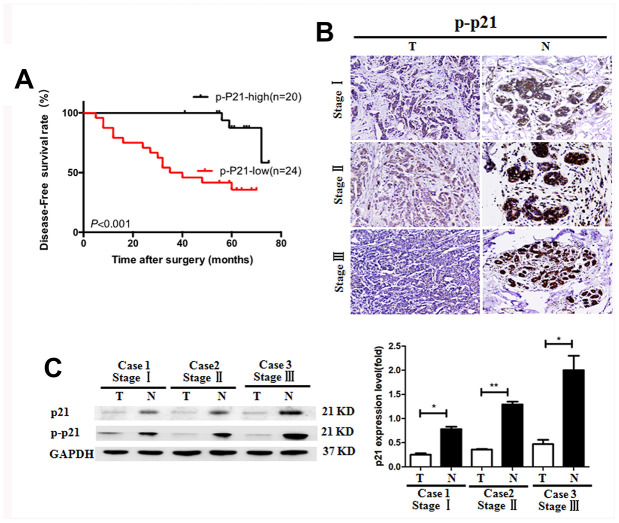
**Reduced p-p21/p21 expression in tumoral tissues of patients with breast cancer.** (**A**) Positive correlation of the disease-free survival rate of patients with the levels of p-p21 expression. (**B**, **C**) P-p21 expression in BC tumoral tissues (T) and BC-adjacent non-tumoral tissues (N) of patients with different clinical stages was evaluated by immunohistochemistry and western blotting. *P < 0.05, **P < 0.01, Equal protein loading in all lanes was confirmed by probing the blots with anti- GAPDH antibody. Magnification: 200×. The experiments were repeated in triplicate.

It was previously reported that the p21 expression was negatively correlated with tumor development in colorectal cancer and gastric carcinoma [23, 24]. However, the role of p21 in the pathogenesis of BC is unclear. In the present study, we found high expression of p-p21 was significantly associated with less lymph node metastasis and early TNM stage (Table 1, p=0.016 and 0.042 respectively). Kaplan-Meier analysis was used to analyze the effect of p-p21 on the survival of BC patients. We showed that the patients with low p-p21 expression had a significantly (p<0.001) shorter disease-free survival (DFS) than those with higher p-p21 expression (Figure 3A). Additionally, multivariate analysis indicated that p-p21 expression was an independent prognostic factor for DFS in the patients (Table 2). Consistent with these results, lower p21 and p-p21 expression was found in the tumoral tissues than in the non-tumoral tissues in BC patients (Figure 3B, 3C), suggesting that p21/p-p21 plays a protective role during the development of BC.

**Table 1:** Correlation between clinicopathological factors and p-p21 expression in patients with breast cancers.

**Factors**	**Cases N=44**	**p-p21**		
		**Low**	**High**	**P value**
Age				
<50	25	11	14	0.107
≥50	19	13	6
TNM stage				
I	17	6	11	0.042*
II+III	27	18	9	
Tumor size				
≤2cm	17	6	11	0.097
>2cm	27	18	9	
Lymph node metastasis				
0	23	8	15	0.016*
1-3	13	9	4	
≥4	8	7	1	
ERα				
Negative	15	9	6	0.601
Positive	29	15	14	
PR				0.152
Negative	9	3	6
Positive	35	21	14
Her-2			
Negative	37	18	19	0.071
Positive	7	6	1	

ER, estrogen receptors; PR, progesterone receptors; HER2, human epidermal growth factor receptor 2. Multinomial logistic regression and likelihood ratio test. The results show that p-p21 expression level were significantly correlated to tumor metastasis and TNM clinical stage (p<0.05). No correlation was detected with gender, age or lymphoid nodal status (p≥0.05).

**Table 2:** Cox regression analysis of baseline predictors of mortality.

**Factors**	**Multivariate**		
	**HR**	**95% CI**	**P value**
p-p21-high	0.112	0.025-0.508	0.005
Her2-high	2.728	1.503-4.950	0.001

HR, hazard ratio; CI, confidence interval.

### C5a/C5aR signaling deficiency prevents the downregulation of the p21 expression in the tumor tissues of mice with BC

**Figure 4:**
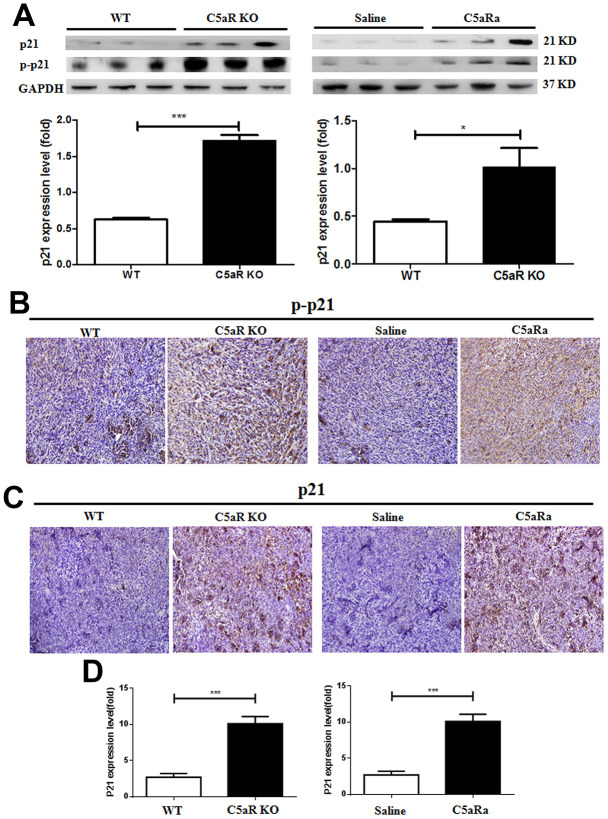
**C5aR deficiency or C5aR signaling blockade inhibits the downregualtion of p21 expression in tumor tissues of mice.** Mice were received 4T-1 cells transplantation for tumor development and sacrificed in the indicated time. (C5aRa treatment group: WT mice were injected with C5aRa 30 min prior to 4T-1 cells transplantation. Saline was used as the control of C5aRa). The tumors tissues were collected. The expression of p21 and p-p21 was measured by western blotting (**A**) and immunohistochemistry (**B**, **C**). (**D**) The mRNA levels of p21 were measured by quantitative RT-PCR. (n=5-6/group). *P < 0.05, ***P < 0.001. GAPDH was used as the loading control. Magnification: 200×.

To investigate the correlation of the C5a/C5aR pathway with p21 expression during BC development, WT and C5aR knock-out mice were transplanted with the BC cell line, 4T-1 cells (p21 positive) (Supplementary Figure 4). The expression of p21 and p-p21 were measured by western blotting, IHC and RT-PCR. The increased expression of the p21 and p-p21 was observed in tumor tissues of mice with C5aR-deficiency or treated with C5aRa (Figure 4A–4D). This suggests that the C5a/C5aR signaling is critical for the reduction of p21 expression during the development of BC.

### C5aR signaling suppresses p21 expression through activation of the MAPK/p38 pathway

**Figure 5:**
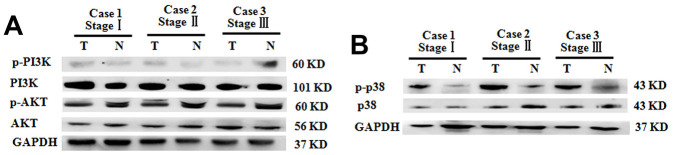
**Increased p38 phosphorylation in BC tumoral tissues of patients with different clinical stages.** (**A**) P-PI3K and p-AKT expression in BC tumoral tissues (T) and BC-adjacent non-tumoral tissues (N). (**B**) Phosphorylation levels of p38 in BC tumoral tissues (T) and BC-adjacent non-tumoral tissues (N). Equal protein loading in all lanes was confirmed by probing the blots with anti- GAPDH antibody. Two independent experiments were performed.

Although the PI3K/AKT signaling pathway was closely related to the genesis and metastasis of some types of tumors, such as colorectal cancer and hepatocellular carcinoma [25, 26], the levels of PI3K/AKT were comparable between the tumoral tissues and para-carcinoma tissue from BC patients with clinical stages (Figure 5A). This is indicative of the non-association of the PI3K/AKT pathway and pathogenesis of BC. In contrast, compared with that in para-carcinoma tissue, the p-p38 levels in the tumoral tissues were significantly increased (Figure 5B).

**Figure 6:**
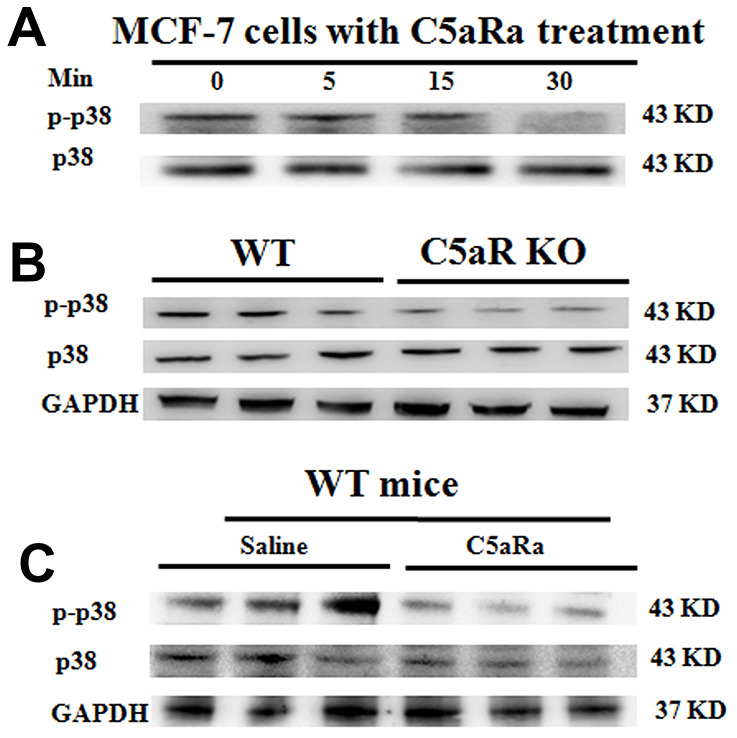
**Reduced p38 phosphorylation in BC tumoral tissues in mice with C5aR deficiency or C5aR signaling blockade.** (**A**) The phosphorylation levels of p38 in human BC cell line MCF-7 cells after C5aRa treatment (10 nM) for indicated times. (**B**, **C**) After transplantation with murine BC cell line 4T-1 cells, mice were sacrificed in the indicated times and tumor tissues were collected (C5aRa treatment group: WT mice was injected with C5aRa 30 min prior to 4T-1 cells transplantation. Saline was used as the control of C5aRa). P-p38/p38 expression levels in tumor tissue were determined by western blotting. GAPDH was used as the loading control. The data are representative of three independent experiments.

To explore the correlation of C5aR signaling with MAPK/p38 pathway activation during BC growth, we treated BC cell line MCF7 cells (which constitutively expressed p21 (Supplementary Figure 4) and C5aR (Supplementary Figure 5A) ) with the C5aRa for 24 h. It was found that p38 phosphorylation levels showed a time-dependent reduction (Figure 6A) as well as cell proliferation was suppressed (Supplementary Figure 5B) and the cells underwent a p21-mediated G2/M phase arrest with a concentration-dependent manner (Supplementary Figure 5C). When mice were transplanted with the BC cell line 4T-1 cells, mice with the C5aR-deficiency or treated with C5aRa exhibited a remarkable decrease in p-p38 levels during the development of BC (Figure 6B, 6C). Taken together, these *in vitro* and *in vivo* results demonstrate that the C5a/C5aR pathway participates in the MAPK/p38 pathway activation during BC development.

**Figure 7:**
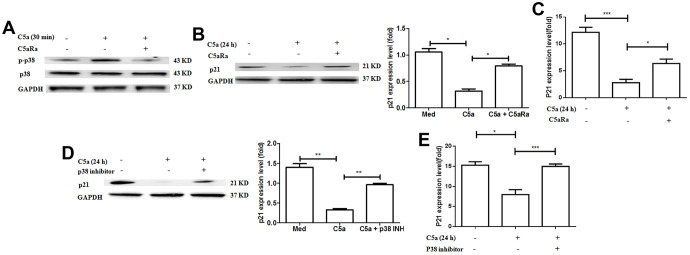
**Involvement of p38 pathway in C5aR signaling-mediated the downregulation of p21 expression.** (**A**–**C**) MCF-7 cells were pre-incubated with C5aRa (10 nM) for 60 min before exposure to recombinant murine C5a (480 ng/mL) for 30 min or 24 h. The cell lysates were assessed by western blotting using a polyclonal antibody against p-p38, p21 and the mRNA levels of p21 were measured by RT-PCR. (**D**, **E**) MCF-7 cells were pre-incubated with p38 inhibitor SB203580 (50 mM) for 60 min before exposure to recombinant murine C5a (480 ng/mL) for 24 h and then p21 expression levels were detected by western blotting with polyclonal antibody against p21 and the mRNA levels of p21 were measured by RT-PCR. *P < 0.05, **P < 0.01, ***P < 0.001. GAPDH was used as the loading control. All *in vitro* measurements were performed in independent quadruplicates with matching results.

To further investigate whether MAPK/p38 is involved in the C5a/C5aR pathway-mediated downregulation of the cell cycle protein p21 in BC, we treated the BC cell line MCF-7 cells with the recombinant C5a and measured the expression of MAPK/p38 and p21. It was observed that the C5a stimulation caused an increased p38 phosphorylation (Figure 7A), but a reduced p21 expression in the BC cells (Figure 7B, 7C), and C5aRa treatment reversed these effects. Moreover, MAPK/p38 inhibitors attenuated C5a-mediated downregulation in p21 expression (Figure 7D, 7E). All these results suggest that the MAPK/p38 pathway participates in the C5a/C5aR-mediated suppression of p21 expression during BC pathogenesis.

## DISCUSSION

The C5a/C5aR pathway has been increasingly recognized as a crucial contributor to cancer progression. Blockade of the C5a/C5aR signaling resulted in an impaired tumor growth in mice [27]. A higher mRNA level of C5aR showed a negative relationship with the survival of ovarian cancer patients and C5a silencing reduced the tumor growth in ovarian cancer cells [28]. In BC, C5aR-positive patients had a lower survival rate than C5aR-negative patients, reiterating that C5aR is a predictor of poor prognosis in BC [13]. Consistently, our data showed that the tumors volume remarkably reduced in C5aR-deficient mice or mice treated with C5aR antagonists, indicating that the C5a/C5aR pathway plays a critical role in the pathogenesis of BC. It was previously showed in a mouse model of BC, that C5a/C5aR system facilitated metastasis in the lungs by creating a microenvironment favourable for cancer cells (by suppressing effector CD8^+^ and CD4^+^ T cell responses) [14]. The reduced tumor growth in mice with pharmacologic blockade of C5aR or its genetic ablation in C5aR was caused by the increase in antitumor immune response, or synergistically inhibiting cancer cell growth by both cancer microenvironment and C5a-C5aR interaction in cancer cells? This needs to be clarified in the future.

The PI3K/AKT pathway participates in a broad range of cell processes, including proliferation, apoptosis, metabolism regulation, and cell cycle progression. Hyper-activation of the PI3K pathway is involved in tumor development [29]. Blocking the PI3K/AKT pathway was beneficial for the clinical treatment of lung cancer [30]. However, our data showed that the MAPK/p38 pathway, and not the PI3K/AKT pathway, was activated, in BC progression. Similar results were observed in other labs. Zheng et al. found that downregulating the phosphorylation of p38/MAPK signaling pathway led to a reduction in the migration of cervical cancer cells [31]. The results of Davidson et al. showed that p38 was expressed in 54/55 (98%) specimens, and its phosphorylated form was found in 51/55 (92%) in effusions from patients diagnosed with serious ovarian carcinoma, suggesting that p38/MAPK signaling was closely related to the incidence of ovarian cancer [32]. The discrepancy is probably contributed to the difference in cancer types. It is reported that p38 is more likely to be activated in women once they develop a tumor since women generally produce high levels of estrogen [33].

P21 is negatively associated with the cancer occurrence [34, 35]. Consistent with these results, we observed that the p21 expression was reduced in the tumor tissues and its low expression was closely associated with poor survival rates in BC patients. In contrast, the data from Yu. et al. showed that the increased expression of p21 caused chemo-resistance in BC cells with HER2 overexpression [36]. P21 overexpression is also associated with the poor prognosis in BC survival [37]. The contradictory functions of the p21 during cancer development may be related to its sub-cellular localization [38]. It was demonstrated that the cytoplasmic localization of p21 was strongly associated with poor prognosis [39–41]. P21 expression in the nucleus promotes tumor cell apoptosis, if expressed in the cytoplasm inhibits its apoptosis. However, the underlying mechanism of p21 expression in BC development needs further exploration in the future.

Collectively, these results reveal that the C5a/C5aR pathway is critical for BC development, for which MAPK/p38 pathway participates in down-regulating p21 expression.

## MATERIALS AND METHODS

### Patients and clinical specimens

BC tissues and tumor-adjacent tissues (5 cm from the tumor margin) were obtained from 44 patients who underwent BC surgery, between 2008 and 2010, with lymph node dissection at the Chongqing Southwest Hospital (China). None of the patients underwent preoperative chemotherapy or radiation therapy. Plasma samples were collected from 27 BC patients and 20 healthy donors. All study procedures were carried out with the pre-approval of the Ethics Committee of the Third Military Medical University. All the patients were informed about the sample processing steps and provided their written consent prior to the study enrollment.

### Cell culture

The human MCF-7 and murine 4T-1 BC cell lines were obtained from the Cell Bank of the Chinese Academy of Sciences (Shanghai, China). They were routinely cultured in Dulbecco’s Modified Eagle’s Medium (high glucose) (Gibco, Life Technologies, USA) supplemented with 10% fetal bovine serum (Gibco, Life Technologies, USA) and maintained at 37 °C in a humidified incubator with 5% CO_2_.

For immunofluorescence staining p21 on 4T-1 and MCF-7 cells, cells were fixed in 4% paraformaldehyde solution for 30 min. After being blocked with 5% goat serum for 30 min, cells were incubated with a mouse anti-mouse/human p21 antibody (1:100 dilution, Beyotime, Shanghai, China) overnight at 4 °C, and then incubated with a Cy-3 goat anti mouse antibody (1:300 dilutions) for 1 h. For immunofluorescence staining C5aR on 4T-1 cells, cells were incubated with a rabbit anti-mouse/human C5aR antibody (1:200 dilutions, Abcam, Cambridge, UK) overnight at 4 °C, and then incubated with a DyLight 488 donkey anti-rabbit IgG antibody (1:300 dilutions, BioLegend, USA) for 1 h. After the nuclei were stained with Hochest33258 (1:300 dilutions) for 2 min, cells were visualized via confocal laser microscope (Leica Microsystem, Germany). For immunocytochemistry staining C5aR on MCF-7 cells, cells were incubated with rabbit anti-mouse/human C5aR antibody (1:100 dilutions) overnight at 4°C and then incubated with horse radish peroxidase (HRP)-labeled anti-rabbit antibodies (1:1000, Beyotime, Shanghai, China) for 60 min at room temperature. Cells were counterstained with hematoxylin and then examined via light microscope (Olympus, Tokyo, Japan).

In order to investigate the role of C5a in PI3K/AKT activation, the MCF-7 cells were stimulated with 480 ng/mL C5a (Biovision, USA) for 30 min. For some assays, the MCF-7 cells were pre-treated with 10 nM C5aRa (GL Biochem, China) for 60 min before stimulation with C5a. To investigate the changes in the p21 expression induced by the C5a stimulation, the MCF-7 cells were incubated for 24 h in the media alone or in the media supplemented with C5a, with or without C5aRa pre-treatment. To determine whether the C5a-mediated impairment of p21 expression is MAPK/p38-dependent, the MCF-7 cells were pre-treated with 50 μM SB203580 (a p38 inhibitor) for 60 min and then stimulated with C5a for 30 min with or without C5aRa pre-treatment. Cells were collected in the indicated time points for western blotting and quantitative reverse transcription-polymerase chain reaction (qRT-PCR) assay.

### Cell cycle

About 5 × 106 human MCF-7 BC cells were harvested at 25 °C after pre-treatment with various concentrations of C5a for 24 h. The supernatant was removed, and the cells were fixed in 70% ice-cold ethanol overnight. Ethanol-fixed cells were re-suspended in 500 μL PBS (PH=7.4) containing 0.1 mg/mL RNase and 40 μg/mL propidium iodide (PI) and were incubated at 37 °C for 30 min. The cell cycle distribution was analyzed using a flow cytometer (BD FACS Canto II, USA).

### Mice studies

Wild-type (WT) BALB/c mice was obtained from the Animal Institute of Academy of Medical Science (Beijing, China). C5aR-deficient mice (BALB/c background) were purchased from Jackson Laboratory (Ellsworth, Maine, US). BALB/c mice were 8-12 weeks old at the beginning of the experiments, and the groups were matched by age and sex. All mice were housed in the individual ventilated cages at the Institute of Immunology of the Third Military Medical University (Chongqing, China). Mice were divided randomly into two groups; both the groups received 4T-1 cells transplantation (~1×10^5^ murine BC cell line 4T-1 cells into the armpit), and one of the two group received an additional injection of the C5aRa (1 mg/kg, intravenously via the tail) 30 min prior to 4T-1 cells transplantation. Mice developed tumors on day 15 after the 4T-1 injection and were sacrificed by retro-orbital bleeding. The tumors tissues were collected immediately and prepared for analysis by immunohistochemistry (IHC), western blotting and qRT-PCR.

### Immunohistochemistry

Serial sections were incubated with a primary antibody against C5aR (rabbit anti-human/mouse at 1:200; Abcam, USA), p21, p-p21 (mouse anti-mouse/human at 1:100; Santa Cruz Biotechnologies, USA), Ki67 (mouse anti-mouse/human at 1:100; Beyotime Biotechnology, China), CD146 (mouse anti-mouse/human at 1:300; Invitrogen, USA) at 4 °C overnight. The method used here has been previously published in detail [42].

### Western blotting

In brief, equal amounts of total protein extracts from the cultured cells or tissues were separated using 8–12% SDS-PAGE and electrotransferred onto the 0.45 μm PVDF membrane. Mouse or rabbit primary antibodies and the respective HRP-conjugated secondary antibodies were used to detect the target proteins. The PVDF membrane was developed using ECL detection reagents (Thermo Scientific) in a dark room. Results were normalized to the internal control GAPDH. Membranes were incubated with a polyclonal rabbit anti-human/mouse C5aR antibody (1:1000; Abcam, USA), a monoclonal mouse anti-mouse p38 antibody (1:1000; Beyotime Biotechnology, China), a monoclonal rabbit anti-mouse p-p38 antibody (1:1000; Beyotime Biotechnology, China), a monoclonal rabbit anti-mouse PI3K/AKT antibody (1:1000; Cell Signaling, USA), a monoclonal rabbit anti-mouse p-PI3K/p-AKT antibody (1:1000; Cell Signaling, USA), a monoclonal mouse anti-mouse/human p21 antibody (1:2000; ZSGB-BIO, China), a monoclonal mouse anti-mouse/human p-p21 antibody (1:500; Santa Cruz Biotechnologies, USA), or a monoclonal mouse anti-mouse/human GAPDH antibody (1:1000; Beyotime Biotechnology, China).

### Enzyme-linked immunosorbent assay

The concentrations of C5 and C5a in the serum samples of the BC patients and healthy volunteers were measured using ELISA kits (USCN, China) targeting various factors.

### Senescence-associated β-galactosidase assay

4T-1 cells treated with 10 nM C5aRa or PBS for 24 h and then fixed with 4% formaldehyde for 20 min. Senescence-associated β-galactosidase (Sen-β-Gal) staining was performed using the Beyotime Biotechnology kit with a modified protocol (Beyotime Biotechnology, C0602). The development of green color was visualized under microscope (Leica, German). Magnification:400 ×.

### Semi-quantitative reverse transcription-polymerase chain reaction (qRT-PCR)

Total RNA of tumor tissues of mice and MCF-7 cells were extracted with RNAiso reagent (Takara, Dalian, China). cDNA were obtained by using a reverse transcription system (Takara, Japan) according to the manufacturer's instructions. Real-time PCR was conducted with an MxPro3000P (Agilent StrataGene, America) and SYBR R Premix Ex TaqTM reagent kit (Takara, Shiga, Japan). Samples were normalized to GAPDH. The primer sequences for human p21: forward 5’-CCGAAGTCAGTTCCTTGTGG-3’, reverse 5’- CATGGGTTCTGACGGACAT -3’. The primer sequences for mouse p21: forward 5’-CCTGGTGATGTCCGACCTG-3’, reverse 5’-CCATGAGCGCATCGCAATC- 3’. The primer sequences for GAPDH: forward 5’-CTCTGCTCCTCCTGTTCGAC -3’, reverse 5’-CTCTGCTCCTCCTGTTCGAC-3’. All experiments were performed in either duplicate or triplicate, and were normalized with respect to GAPDH levels.

### *in vitro* breast cancer cells migration and invasion assay

For transwell migration assays, 2.5 ~ 5 × 10^4^ non-adherent BC cell line MDA-MB-453 cells were plated in the top chamber with the non-coated membrane (24-well insert; pore size, 8 μm; BD Biosciences). For invasion assays, 1.25 × 10^5^ cells were plated in the top chamber with Matrigel-coated membrane (24-well insert; pore size, 8 μm; BD Biosciences). In both assays, cells were plated in medium without serum, and medium supplemented with 480 ng/mL recombinant C5a were used as a chemoattractant in the lower chamber. Cells were incubated for 12 h and cells that did not migrate or invade through the pores were removed by a cotton swab. Cells on the lower surface of the membrane were stained with the crystal violet dye and counted. The results were observed under the microscope. Magnification: 200 ×.

### Statistical analysis

Differences between groups were evaluated by t test, as specified in the figure legends, by using GraphPad prism Software (La Jolla, CA, USA). Significance was assumed at a p value of 0.05 or less.

### Availability of data and materials

The data are fully available without restriction.

### Ethics approval and consent to participate

Clinical data have been approved by the Ethics Committee of the Third Military Medical University and approved by the patients. All animal experiments were approved by Animal Care and Use Committee of Third Military Medical University.

### Consent for publication

All authors agree to the publication of this article.

## Supplementary Material

Supplementary Figures
