# Supplementary material for: C5aR deficiency attenuates the breast cancer development via the p38/p21 axis
Source: Aging (Albany NY). 2020 Jul 15;12(14):14285–99. doi: 10.18632/aging.103468 (PMC7425439; doi:10.18632/aging.103468)
Supplement: Supplementary Figures [file aging-12-103468-s001..pdf]

SUPPLEMENTARY FIGURES

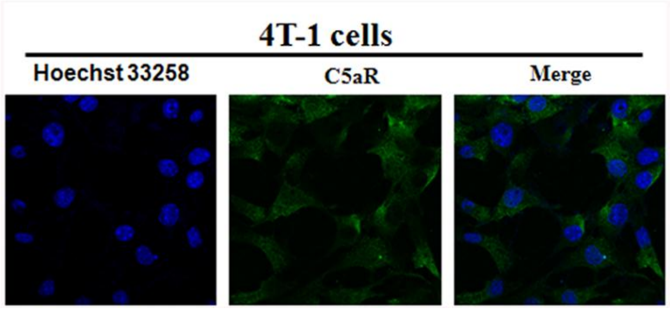

Supplementary Figure 1. Expression of C5aR on 4T-1 cells. The C5aR expression on 4T-1 cells was determined by confocal laser scanning microscopy. Nuclei were stained with Hoechst 33258 solution. Magnification: 400×.

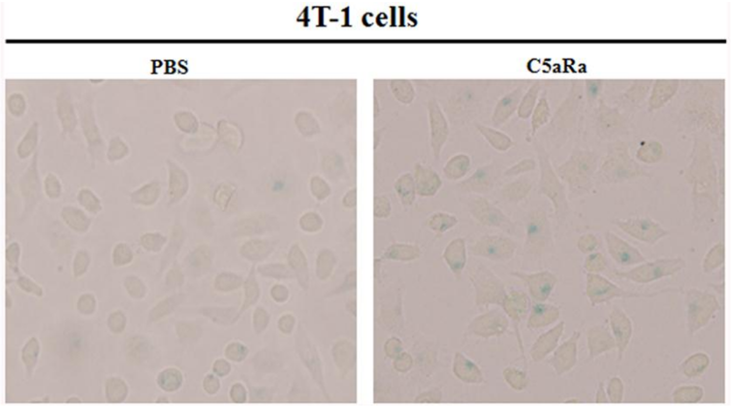

Supplementary Figure 2. Increased aging of BC cells after treatment with C5aR antagonists. 4T-1 cells were treated with C5aRa (10 nM) or PBS for 24 h. Senescence -associated β-galactosidase (Sen-β-Gal) staining was performed using the Beyotime Biotechnology kit. Magnification: 400×.

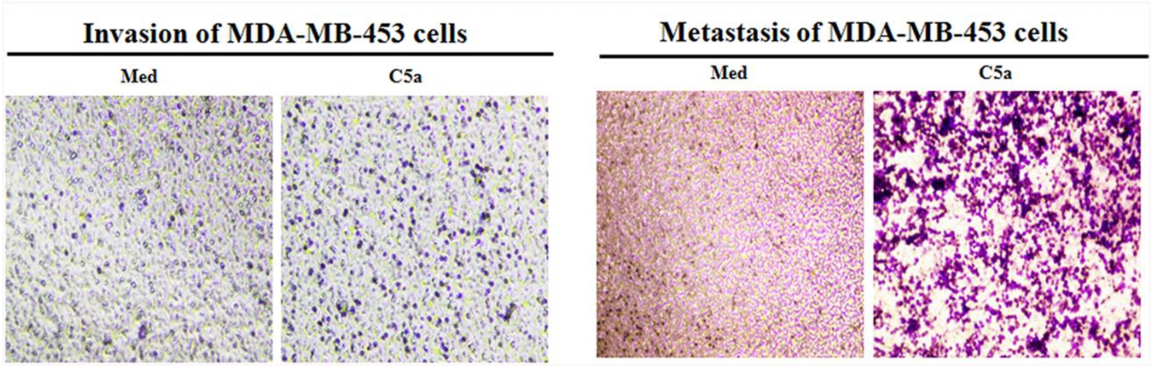

Supplementary Figure 3. Increased cell invasion and metastasis of BC cells after exposure to C5a. Non-adherent BC cell line MDA-MB-453 cells were treated with recombinant C5a (480 ng/mL) for 12 h and cells migration and invasion were assayed. Magnification: 200×.

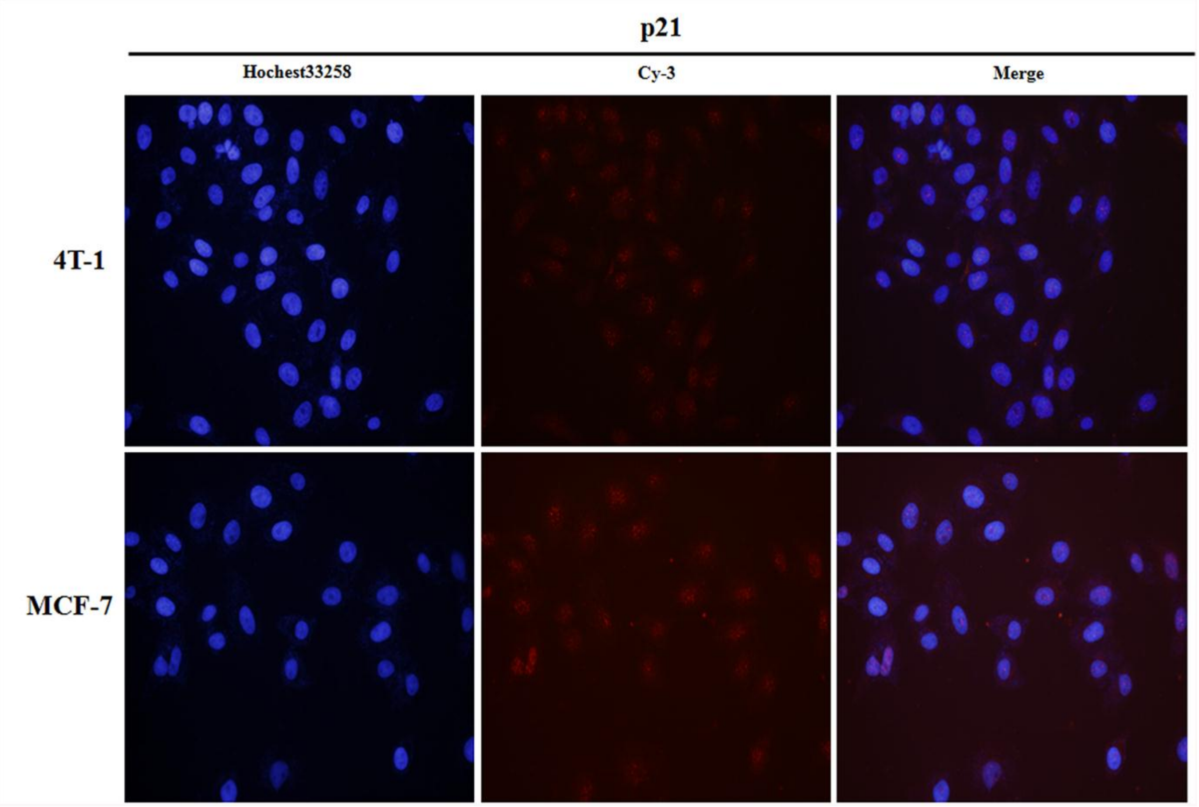

Supplementary Figure 4. Expression of p21 on 4T-1 and MCF-7 cells. The p21 expression was determined by confocal laser scanning microscopy. Nuclei were stained with Hoechst 33258 solution. Magnification: 400×.

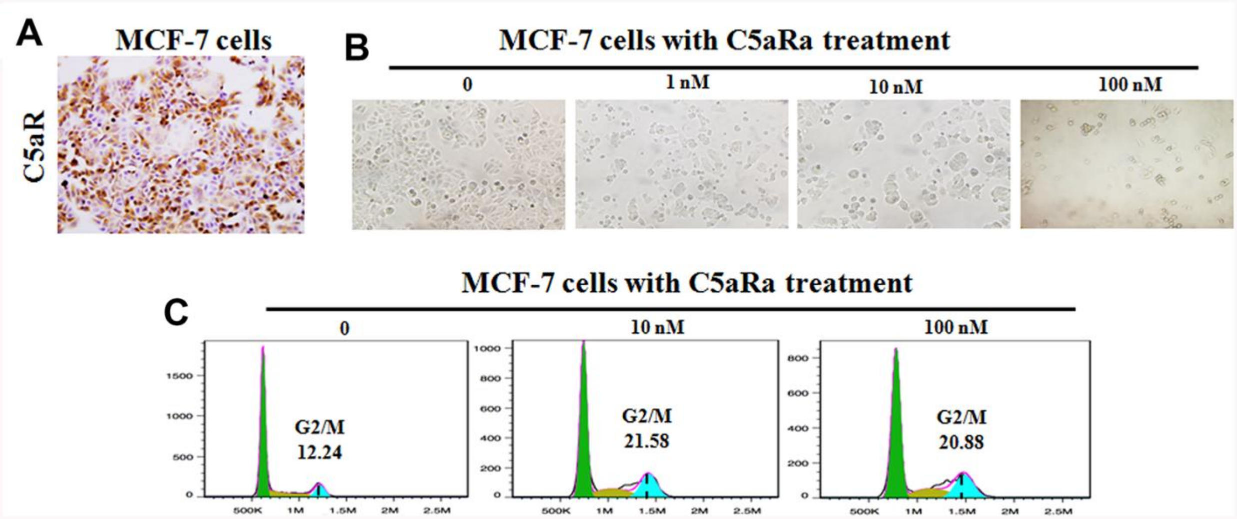

Supplementary Figure 5. C5aRa treatment down-regulates the proliferation of BC cell line MCF-7 cells. (**A**) Constitutive expression of C5aR. (**B**) Cells proliferation was showed and (**C**) Cells cycle distribution was assessed by flow cytometry after cells were incubated with 0, 1, 10, and 100 nM C5aRa for 24 h. Magnification: 200×.
